# Supplementary material for: HIVprotI: an integrated web based platform for prediction and design of HIV proteins inhibitors
Source: J Cheminform. 2018 Mar 9;10:12. doi: 10.1186/s13321-018-0266-y (PMC5845081; doi:10.1186/s13321-018-0266-y)
Supplement: Supplementary file 2 — Additional file 2. Source code of HIVProtI web server. [file 13321_2018_266_MOESM2_ESM.zip › HIVprotI_Source-code/hivprotI/weka-3-6-6/doc/weka/classifiers/bayes/net/search/global/package-summary.html]

weka.classifiers.bayes.net.search.global


---


|  |  |  |
| --- | --- | --- |
| **Overview** \| **Package** \| Class \| **Tree** \| **Deprecated** \| **Index** \| **Help** \| **Weka's home** | |  |
| **PREV PACKAGE**   **NEXT PACKAGE** | **FRAMES**    **NO FRAMES**     **All Classes** |  |


---

## Package weka.classifiers.bayes.net.search.global

| **Class Summary** | |
| --- | --- |
| **GeneticSearch** | This Bayes Network learning algorithm uses genetic search for finding a well scoring Bayes network structure. |
| **GlobalScoreSearchAlgorithm** | This Bayes Network learning algorithm uses cross validation to estimate classification accuracy. |
| **HillClimber** | This Bayes Network learning algorithm uses a hill climbing algorithm adding, deleting and reversing arcs. |
| **K2** | This Bayes Network learning algorithm uses a hill climbing algorithm restricted by an order on the variables.    For more information see:    G.F. |
| **RepeatedHillClimber** | This Bayes Network learning algorithm repeatedly uses hill climbing starting with a randomly generated network structure and return the best structure of the various runs. |
| **SimulatedAnnealing** | This Bayes Network learning algorithm uses the general purpose search method of simulated annealing to find a well scoring network structure.    For more information see:    R.R. |
| **TabuSearch** | This Bayes Network learning algorithm uses tabu search for finding a well scoring Bayes network structure. |
| **TAN** | This Bayes Network learning algorithm determines the maximum weight spanning tree and returns a Naive Bayes network augmented with a tree.    For more information see:    N. |

---


|  |  |  |
| --- | --- | --- |
| **Overview** \| **Package** \| Class \| **Tree** \| **Deprecated** \| **Index** \| **Help** \| **Weka's home** | |  |
| **PREV PACKAGE**   **NEXT PACKAGE** | **FRAMES**    **NO FRAMES**     **All Classes** |  |


---
